# Data on winged insect dynamics in melon crops in southeastern France

**DOI:** 10.1016/j.dib.2020.105132

**Published:** 2020-01-14

**Authors:** Alexandra Schoeny, Patrick Gognalons

**Affiliations:** INRAE, Pathologie Végétale, F-84140, Montfavet, France

**Keywords:** Aphid communities, Biodiversity, Insect sampling, Species composition and population dynamic, Suction trap, Taxonomic identification, Temporal pattern of flight activity, Yellow pan trap

## Abstract

This article displays insect count data obtained in eleven field trials conducted between 2010 and 2019 in southeastern France. Winged insect abundances were monitored daily within melon crops during 8–11 weeks in May–July using a suction trap or a yellow pan trap. Aphids were identified under a stereomicroscope. In total, 29,709 winged aphids belonging to 216 taxa and 151,061 other flying insects were caught. Among possible uses, these data can populate larger multisite studies or larger time series investigating aphid community variations. They can also feed generic studies exploring temporal dependencies or species assemblages. They can stimulate new collaborations with entomologists keen on implementing molecular tools or taxonomic expertise on a large specimen collection.

**Table:** Specifications Table

Subject	Agricultural and Biological Sciences
Specific subject area	Entomology
Type of data	Tables and figures
How data were acquired	Winged aphids were identified under a stereomicroscope using taxonomic keys.
Data format	Raw and summarized
Parameters for data collection	Eleven field experiments were conducted between 2010 and 2019. Winged insects were monitored daily within melon crops during 8–11 weeks in May–July.
Description of data collection	Winged insects were sampled at the crop height with a suction trap placed in the crop. For three of the trials, an additional sampling was carried out with a yellow pan trap. Both types of catches were collected daily, rinsed and stored in 70% ethanol until sorting (aphids vs other insects) and taxonomic identification of aphids under a stereomicroscope.
Data source location	INRAE St Paul experimental station, Avignon, France 43°54′53N, 4°52′59E 43.9147222, 4.8830555 INRAE St Maurice experimental station, Montfavet Avignon, France 43°56′49N, 4°51′52E 43.9469444, 4.8644444
Data accessibility	Summarized data are hosted with the article. Raw data are hosted in a public repository Repository name: Data INRAE (Dataverse) Data identification number: 10.15454/NKRWEO Direct URL to data: https://doi.org/10.15454/NKRWEO

**Table:** 

**Value of the Data** • These daily abundances are useful to characterize and compare the temporal patterns of 206 aphid taxa visiting melon plants cultivated in one geographical area (Avignon) during eight cropping seasons. • These data can benefit other scientists keen to add Avignon datasets in a multisite analysis focusing on a particular aphid taxon or interested in species richness and diversity. They can also populate larger time series investigating community assemblage variations in a context of climate change for instance. • The data can feed generic studies exploring temporal dependencies or species assemblages. • The data can be useful to compare different insect trapping methods and could stimulate other teams to develop the suction trap described in this paper. • Most data correspond to stored specimens that could be shared with entomologists interested in the taxonomic identification of non-aphid taxa, or the implementation of molecular tools to genotype a given taxon or identify a particular gene (insecticide resistance for instance).

## 1 Data

Table 1 presents the melon crop details for each of the 11 field trials: location, date of planting, trial area, number of plants, number of rows, number of plants per row, row spacing and plant spacing.

**Table 1:** Melon crop details for field trials conducted in Avignon between 2010 and 2019.

Trial code	Experimental site	Planting date	Trial area (m^2^)	Number of plants	Number of rows	Number of plants per row	Row spacing (m)	Plant spacing (m)
M10	St Paul	28/05/2010	256	160	8	20	2	0.8
V11	St Paul	09/05/2011^a^	120	120	6	20	2	0.5
V12	St Paul	11/05/2012^a^	150	150	6	25	2	0.5
V13	St Paul	06/05/2013^a^	150	150	6	25	2	0.5
P11	St Paul	24/05/2011	156	208	16	13	1.5	0.5
P12	St Paul	31/05/2012	180	240	16	15	1.5	0.5
P13	St Paul	24/05/2013	180	240	16	15	1.5	0.5
P14	St Paul	27/05/2014	180	240	16	15	1.5	0.5
P15	St Paul	28/05/2015	180	240	16	15	1.5	0.5
M18	St Maurice	25/05/2018	120	160	8	20	1.5	0.5
M19	St Maurice	28/05/2019	120	160	8	20	1.5	0.5

a Agryl P17 fleece removal; fleece optimizes plant growth by increasing both air and soil temperatures and reducing wind damage.

Table 2 presents the 216 aphid taxa recorded during the insect monitoring conducted in Avignon between 2010 and 2019.

**Table 2:** List of aphid taxa recorded during the 2010–2019 monitoring and their corresponding INRA or Rothamsted Insect Survey (RIS) codes.

Taxon name	Taxon code
*Acyrthosiphon caraganae*	RIS-755
*Acyrthosiphon loti*	RIS-381
*Acyrthosiphon malvae*	RIS-382
*Acyrthosiphon pisum*	RIS-389
*Acyrthosiphon primulae*	RIS-392
*Acyrthosiphon* spp	RIS-1014
*Adelges* spp	RIS-2065
*Amphorophora rubi*	RIS-468
*Anoecia corni*	RIS-480
*Anoecia* spp	RIS-1012
*Anuraphis farfarae*	RIS-238
*Anuraphis* spp	RIS-1015
*Anuraphis subterranea*	RIS-239
*Aphis (Protaphis) anuraphoides*	INRA-001
*Aphis (Protaphis) spp*	RIS-1064
*Aphis (Protaphis) terricola*	INRA-002
*Aphis armoraciae*	INRA-003
*Aphis craccivora*	RIS-163
*Aphis fabae*	RIS-132
*Aphis gossypii*	RIS-181
*Aphis nasturtii*	RIS-152
*Aphis nerii*	RIS-787
*Aphis pomi*	RIS-153
*Aphis salicariae*	RIS-142
*Aphis sambuci*	RIS-125
*Aphis* spp	RIS-1005
*Aphis verbasci*	RIS-197
*Aploneura lentisci*	RIS-530
*Appendiseta robiniae*	RIS-793
*Aspidaphis adjuvans*	RIS-260
*Atheroides serrulatus*	RIS-59
*Aulacorthum solani*	RIS-376
*Aulacorthum speyeri*	RIS-377
*Baizongia pistaceae*	RIS-531
*Betulaphis quadrituberculata*	RIS-84
*Brachycaudus cardui*	RIS-241
*Brachycaudus helichrysi*	RIS-243
*Brachycaudus populi*	RIS-747
*Brachycaudus rumexicolens*	RIS-253
*Brachycaudus schwartzi*	RIS-745
*Brachycaudus sedi*	RIS-254
*Brachycaudus* spp	RIS-1016
*Brachycaudus tragopogonis*	RIS-252
*Brachycolus cucubali*	RIS-262
*Brevicoryne brassicae*	RIS-264
*Calaphis flava*	RIS-82
*Callipterinella minutissima*	RIS-80
*Capitophorus carduinus*	RIS-341
*Capitophorus elaeagni*	RIS-342
*Capitophorus hippophaes*	RIS-343
*Capitophorus horni*	RIS-344
*Capitophorus similis*	RIS-346
*Capitophorus* spp	RIS-1018
*Cavariella aegopodii*	RIS-292
*Cavariella archangelicae*	RIS-293
*Cavariella* spp	RIS-1046
*Cavariella theobaldi*	RIS-298
*Ceruraphis eriophori*	RIS-211
*Chaetosiphon fragaefolii*	RIS-287
*Chaetosiphon tetrarhodum*	RIS-289
*Chaitophorus leucomelas*	RIS-50
*Chaitophorus populeti*	RIS-45
*Chaitophorus populialbae*	RIS-46
*Chaitophorus salicti*	RIS-47
*Chaitophorus* spp	RIS-1002
*Chromaphis juglandicola*	RIS-61
*Chromaphis* spp	RIS-1078
*Clethrobius comes*	RIS-87
*Coloradoa rufomaculata*	RIS-280
*Coloradoa* spp	RIS-1020
*Coloradoa tanacetina*	RIS-281
*Corylobium avellanae*	RIS-403
*Cryptomyzus ribis*	RIS-340
*Ctenocallis setosus*	RIS-77
*Diuraphis (Holcaphis) spp*	RIS-1502
*Diuraphis muehlei*	RIS-259
*Diuraphis noxia*	RIS-809
*Drepanosiphum platanoidis*	RIS-91
*Dysaphis plantaginea*	RIS-234
*Dysaphis pyri*	RIS-235
*Dysaphis* spp	RIS-1006
*Ericaphis ericae*	RIS-284
*Eriosoma lanigerum*	RIS-497
*Eriosoma* spp	RIS-1010
*Eriosoma ulmi*	RIS-500
*Essigella californica*	INRA-005
*Essigella* spp	RIS-1518
*Eucallipterus tiliae*	RIS-70
*Eucarazzia elegans*	RIS-768
*Euceraphis punctipennis*	RIS-88
*Forda formicaria*	RIS-527
*Geoica setulosa*	RIS-532
*Geoica* spp	RIS-1055
*Hayhurstia atriplicis*	RIS-261
*Hayhurstia* spp	RIS-1022
*Hoplocallis pictus*	RIS-772
*Hyadaphis coriandri*	RIS-808
*Hyadaphis foeniculi*	RIS-271
*Hyadaphis* spp	RIS-1023
*Hyalopteroides humilis*	RIS-276
*Hyalopterus pruni*	RIS-110
*Hyalopterus* spp	RIS-1065
*Hyperomyzus lactucae*	RIS-358
*Hyperomyzus lampsanae*	RIS-359
*Hyperomyzus pallidus*	RIS-360
*Hyperomyzus picridis*	RIS-362
*Hyperomyzus* spp	RIS-1007
*Illinoia goldamaryae*	RIS-475
*Lipaphis erysimi*	RIS-267
*Macchiatiella rhamni*	INRA-007
*Macrosiphoniella absinthii*	RIS-451
*Macrosiphoniella oblonga*	RIS-461
*Macrosiphoniella persequens*	RIS-462
*Macrosiphoniella sanborni*	RIS-456
*Macrosiphoniella* spp	RIS-1027
*Macrosiphoniella tapuskae*	RIS-732
*Macrosiphum euphorbiae*	RIS-410
*Macrosiphum rosae*	RIS-416
*Macrosiphum* spp	RIS-1009
*Megoura viciae*	RIS-470
*Melanaphis bambusae*	RIS-811
*Melanaphis luzulella*	RIS-122
*Melanaphis pyraria*	RIS-727
*Metopolophium albidum*	RIS-395
*Metopolophium dirhodum*	RIS-396
*Metopolophium festucae*	RIS-397
*Metopolophium frisicum*	RIS-398
*Metopolophium* spp	RIS-1008
*Microlophium* spp	RIS-2014
*Mimeuria ulmiphila*	RIS-510
*Mindarus abietinus*	RIS-491
*Monelliopsis caryae*	RIS-801
*Myzocallis castanicola*	RIS-63
*Myzocallis coryli*	RIS-64
*Myzocallis komareki*	INRA-009
*Myzocallis occidentalis*	INRA-010
*Myzocallis* spp	RIS-1003
*Myzotoxoptera* spp	RIS-1077
*Myzotoxoptera wimshurstae*	RIS-364
*Myzus cerasi*	RIS-312
*Myzus ligustri*	RIS-320
*Myzus lythri*	RIS-314
*Myzus ornatus*	RIS-315
*Myzus persicae*	RIS-322
*Myzus* spp	RIS-1030
*Myzus varians*	RIS-740
*Nasonovia pilosellae*	RIS-354
*Nasonovia ribisnigri*	RIS-355
*Nasonovia* spp	RIS-1011
*Nearctaphis bakeri*	RIS-733
*Ovatus insitus*	RIS-303
*Ovatus* spp	RIS-1025
*Paracletus cimiciformis*	RIS-525
*Pemphigus* spp	RIS-1506
*Phorodon cannabis*	RIS-812
*Phorodon humuli*	RIS-308
*Phylloxera* spp	RIS-2003
*Pleotrichophorus glandulosus*	RIS-350
*Pseudacaudella rubida*	RIS-275
*Pterocallis alni*	RIS-75
*Rhodobium porosum*	RIS-401
*Rhopalomyzus poae*	RIS-309
*Rhopalosiphoninus ribesinus*	RIS-367
*Rhopalosiphum insertum*	RIS-111
*Rhopalosiphum maidis*	RIS-112
*Rhopalosiphum nymphaeae*	RIS-113
*Rhopalosiphum padi*	RIS-114
*Rhopalosiphum rufiabdominale*	RIS-2009
*Rhopalosiphum rufulum*	RIS-739
*Rhopalosiphum* spp	RIS-1045
*Schizaphis graminum*	RIS-116
*Schizaphis palustris*	RIS-115
*Schizaphis pilipes*	RIS-750
*Schizaphis scirpi*	RIS-121
*Semiaphis dauci*	RIS-728
*Semiaphis* spp	RIS-1088
*Sipha elegans*	RIS-52
*Sipha maydis*	RIS-54
*Sitobion avenae*	RIS-420
*Sitobion fragariae*	RIS-421
*Sitobion* spp	RIS-1031
*Smynthurodes betae*	RIS-526
*Staegeriella necopinata*	RIS-273
*Subsaltusaphis picta*	RIS-738
*Taiwanaphis* spp	INRA-012
*Takecallis arundicolens*	RIS-72
*Takecallis arundinariae*	RIS-73
*Takecallis taiwanus*	RIS-74
*Tetraneura nigriabdominalis*	RIS-2008
*Tetraneura* spp	RIS-1037
*Tetraneura ulmi*	RIS-503
*Thelaxes dryophila*	RIS-490
*Thelaxes* spp	RIS-1038
*Therioaphis luteola*	RIS-92
*Therioaphis ononidis*	RIS-93
*Therioaphis riehmi*	RIS-731
*Therioaphis* spp	RIS-1039
*Therioaphis trifolii*	RIS-94
*Tinocallis kahawaluokalani*	RIS-795
*Tinocallis takachihoensis*	RIS-797
*Tuberculatus (Tuberculoides)* spp	RIS-1024
*Tuberculatus annulatus*	RIS-68
*Tuberculatus borealis*	RIS-758
*Tuberculatus neglectus*	RIS-759
*Tuberculatus querceus*	RIS-69
*Tuberolachnus salignus*	RIS-23
*Uroleucon (Uroleucon)* spp	INRA-015
*Uroleucon (Uromelan)* spp	RIS-1504
*Uroleucon ambrosiae*	INRA-013
*Uroleucon compositae*	INRA-014
*Uroleucon erigeronense*	RIS-763
*Uroleucon tussilaginis*	RIS-439
*Utamphorophora humboldti*	RIS-751
*Wahlgreniella nervata*	RIS-782
*Wahlgreniella* spp	RIS-1042
*Wahlgreniella vaccinii*	RIS-479

Table 3 presents a summary of airborne insect monitoring in 11 field trials conducted in Avignon between 2010 and 2019. In total, 29,709 winged aphids and 151,061 other flying insects were caught. According to the dataset, the abundance of winged aphids varied between 431 and 4206; the abundance of other flying insects varied between 1169 and 23,139. Per dataset, aphids represented between 5 and 35% of the catch. Between 35 and 107 aphid taxa were recorded per dataset. A small proportion of aphids (0.3–2.5% per dataset) could not be assigned to a taxon because of i) limit of taxonomic expertise, ii) loss during storage, or iii) damage during trapping.

**Table 3:** Summary of airborne insect monitoring in 11 field trials conducted in Avignon between 2010 and 2019.

Dataset code	Trial code	Trapping method	Monitoring period (days)	Number of winged aphids	Number of other flying insects	Ratio aphids/total catch (%)	Number of aphid taxa identified	Number of aphids not assigned to a taxon^a^
M10	M10	Suction	64	3532	14 871	19	107	81
V11	V11	Suction	74	3128	16 423	16	92	13
V12	V12	Suction	66	4206	23 139	15	95	106
V13	V13	Suction	80	2998	13 488	18	99	17
P11	P11	Suction	65	3306	17 924	16	91	19
P12	P12	Suction	56	3602	11 499	24	75	57
P13	P13	Suction	62	1848	7571	20	80	5
P14	P14	Suction	59	1457	9346	13	62	7
P15	P15	Suction	56	2245	7825	22	51	18
P15Y	P15	Yellow pan	56	518	1169	31	35	8
M18	M18	Suction	55	786	15 660	5	81	4
M18Y	M18	Yellow pan	55	431	2132	17	49	5
M19	M19	Suction	58	835	8476	9	76	6
M19Y	M19	Yellow pan	58	817	1538	35	52	7
MIN			55	431	1169	5	35	4
MAX			80	4206	23 139	35	107	106
TOTAL				29 709	151 061			

a Aphids that could not be identified because of i) limit of taxonomic expertise, ii) loss during storage, or iii) damage during trapping.

**Fig. 1:**
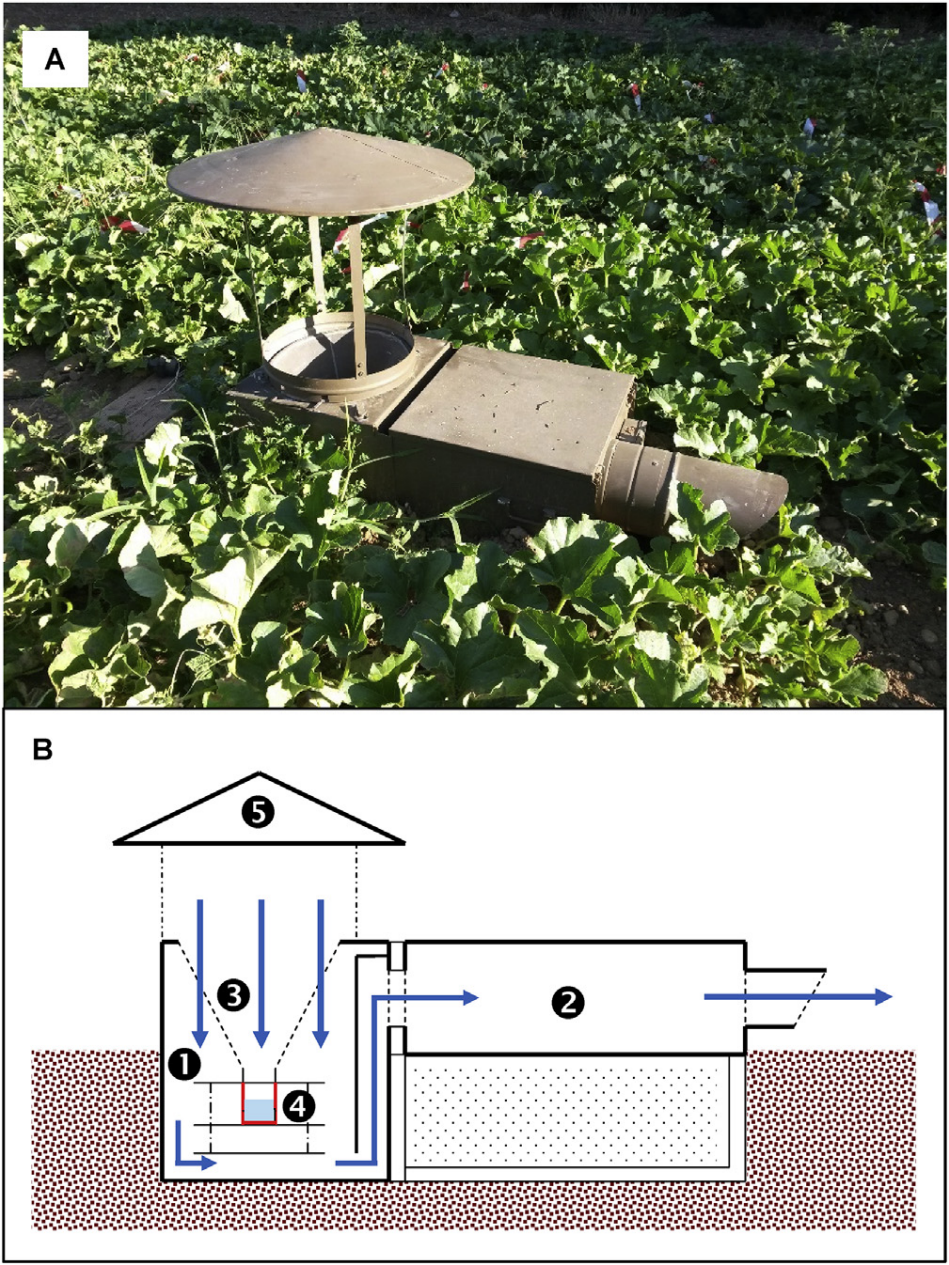
Suction trap used to monitor winged insects in eleven field trials conducted in Avignon between 2010 and 2019. (A) In situ in a melon crop (Photo credit: Alexandra Schoeny, INRAE) (B) Schematic representation of a suction trap adapted from Pascal et al., 2013 [2] showing its operating principle and its different parts: ➊ vacuum chamber, ➋ air extractor, ➌ insect collector, ➍ collecting pot, ➎ chimney rain cap.

Fig. 1 illustrates the main trapping method used to monitor winged insects in each of the 11 trials (suction trap).

**Fig. 2:**
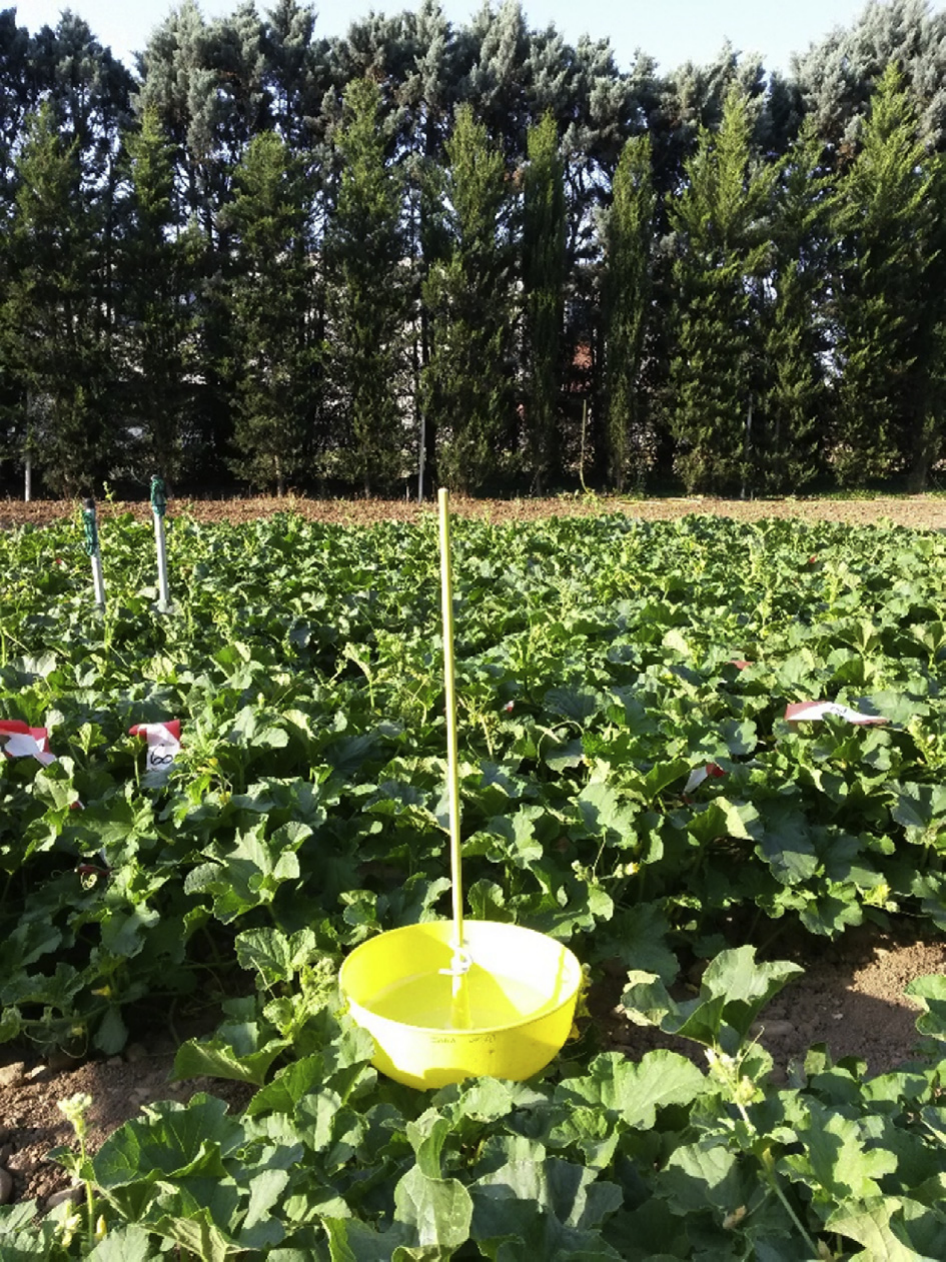
Yellow pan trap used to monitor winged insects in three of the eleven field trials conducted in Avignon between 2010 and 2019 (Photo credit: Alexandra Schoeny, INRAE).

Fig. 2 illustrates a complementary trapping method (yellow pan trap) used in three of the 11 trials.

## 2 Experimental design, materials, and methods

1. Field experiments

Eleven field experiments were conducted between 2010 and 2019 at INRAE Avignon, southeastern France: nine at St Paul experimental station (43°54′53N, 4°52′59E) and two at St Maurice experimental station (43°56′49N, 4°51′52E) (Table 1). The two sites are approximately 4 km apart and surrounded by a highly diversified environment consisting of discontinuous urban fabric, commercial units, arable land, permanent crops (vineyards, fruit trees, olive groves), pastures and mixed forest, according to CORINE land cover nomenclature [1].

The experimental design consisted of a Charentais-type melon crop which layout varied according to trials (Table 1). Seedlings were prepared in an insect-proof greenhouse three weeks before planting. Depending on the trial, plants at the 1–3 leaf stage were planted in late April or late May on dark brown plastic mulch with drip irrigation. Early plantings were protected from wind damage with Agryl P17 fleece (Fiberweb France, Biesheim) for 11–15 days. The crop comprised 120 to 240 plants (0.5–0.8 m plant spacing) organized in 6–16 rows (1.5–2 m row spacing) depending on the trial. No insecticides were applied during trials.

2. Insect trapping and winged aphid identification

A non-biased suction trap was designed to sample winged insects daily at the crop height [2]. It is made up of a vacuum chamber generating a downward suction, an air extractor (400 m^3^/h, 160B model, France Air), an insect collector and a chimney rain cap (Fig. 1). The insect collector is inserted in the vacuum chamber. Small insects flying above the vacuum chamber opening are catched and dragged in a collecting pot containing 100 ml of water with 5 μl/l detergent (Teepol 610 S, ref 86350, Sigma-Aldrich) to break the surface tension and prevent insects from escaping. Each trial was equipped with a suction trap set up in the melon crop. The trap runned daily for a 12-h sequence (8:00 a.m. - 8:00 p.m.) thanks to a timer. The collecting pot was changed daily before the start of the trapping.

For three of the 11 field trials, winged insects were also sampled with a yellow pan trap (model FLORA cultures basses, ref 058501, SigneNature) placed at 2–3 m from the suction trap (Fig. 2). The trap was filled with 1 l of water with 5 μl/l detergent and changed daily at 8:00 a.m.

Airborne insect monitoring started at crop planting or fleece removal to avoid bias caused by a possible visual repellent effect of the fleece on winged aphid behaviour. Depending on the trial, it was carried out for 55–80 days. Catches were collected daily, rinsed with tap water and stored in 70% ethanol until sorting (aphids vs other insects) and taxonomic identification (aphids only) under a stereomicroscope. Aphids were identified based on morphological characteristics using several dichotomous keys [[3], [4], [5]] and counted. Individuals which could not be identified to species were grouped at genus level.

Whenever possible, aphid species/genera were associated with their Rothamsted Insect Survey (RIS) codes (Table 2). For aphid taxa not yet referenced in the RIS system, INRA codes were assigned.

## References

[1] Enhanced CLC nomenclature guidelines. https://land.copernicus.eu/user-corner/technical-library/corine-land-cover-nomenclature-guidelines/html/.

[2] Pascal F., Bastien J.M., Schoeny A. (2013). Fabrication d'un piège à aspiration pour la capture des pucerons ailés vecteurs de virus. Le Cahier des Techniques de l'Inra.

[3] Blackman R.L., Eastop V.F. (2000). Aphids on the World's Crops: an Identification and Information Guide.

[4] Remaudière G., Seco Fernandez M.V. (1990). Claves para ayudar al reconocimiento de alados de pulgones trampeados en la region mediterranea (Homoptera Aphidoidea).

[5] Taylor L.R. (1984). A Handbook for Aphid Identification.

